# Targeting TSPO Reduces Inflammation and Apoptosis in an In Vitro Photoreceptor-Like Model of Retinal Degeneration

**DOI:** 10.1021/acschemneuro.2c00582

**Published:** 2022-10-27

**Authors:** Francesca Corsi, Emma Baglini, Elisabetta Barresi, Silvia Salerno, Chiara Cerri, Claudia Martini, Federico Da Settimo Passetti, Sabrina Taliani, Claudia Gargini, Ilaria Piano

**Affiliations:** Department of Pharmacy, University of Pisa, Pisa 56126, Italy

**Keywords:** (max 6), translocator protein (TSPO), inflammation, neurodegeneration, photoreceptor cell line, *N*,*N*-dialkyl-2-arylindol-3-ylglyoxylamides (PIGAs)

## Abstract

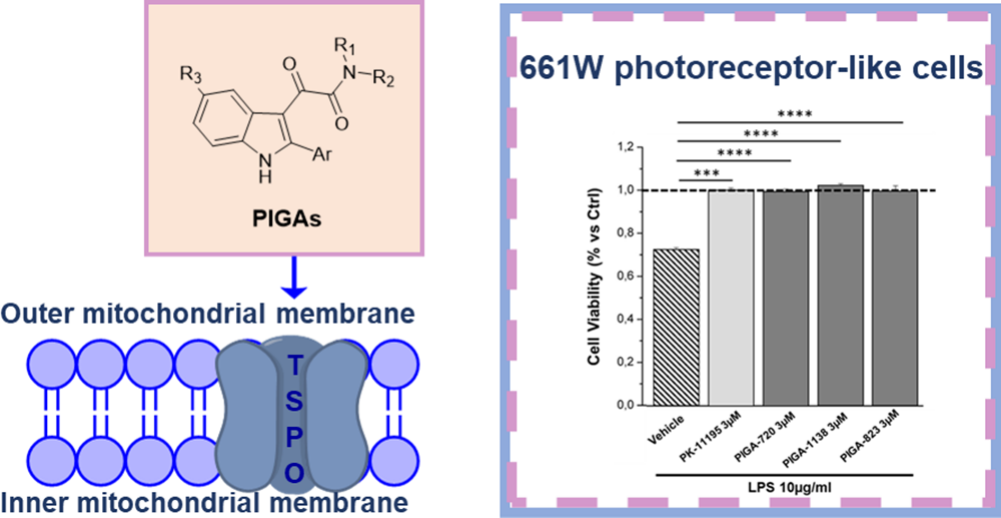

The 18 kDa translocator protein (TSPO) is predominantly located in the mitochondrial outer membrane, playing an important role in steroidogenesis, inflammation, survival, and cell proliferation. Its expression in the CNS, and mainly in glial cells, is upregulated in neuropathologies and brain injury. In this study, the potential of targeting TSPO for the therapeutic treatment of inflammatory-based retinal neurodegeneration was evaluated by means of an in vitro model of lipopolysaccharide (LPS)-induced degeneration in 661 W cells, a photoreceptor-like cell line. After the assessment of the expression of TSPO in 661W cells, which, to the best of our knowledge, was never investigated so far, the anti-inflammatory and cytoprotective effects of a number of known TSPO ligands, belonging to the class of *N*,*N*-dialkyl-2-arylindol-3-ylglyoxylamides (PIGAs), were evaluated, using the classic TSPO ligand PK11195 as the reference standard. All tested PIGAs showed the ability to modulate the inflammatory and apoptotic processes in 661 W photoreceptor-like cells and to reduce LPS-driven cellular cytotoxicity. The protective effect of PIGAs was, in all cases, reduced by cotreatment with the pregnenolone synthesis inhibitor SU-10603, suggesting the involvement of neurosteroids in the protective mechanism. As inflammatory processes play a crucial role in the retinal neurodegenerative disease progression toward photoreceptors’ death and complete blindness, targeting TSPO might represent a successful strategy to slow down this degenerative process that may lead to the inexorable loss of vision.

## Introduction

The 18 kDa translocator protein (TSPO) is a highly conserved nuclear-encoded protein primarily localized in the outer mitochondrial membrane.^1^ TSPO is involved in numerous cellular functions related to the regulation of mitochondrial cholesterol translocation, porphyrin transport and heme synthesis,^2^ cell proliferation,^3^ apoptosis,^4,5^ immunomodulation,^6^ stress adaptation,^7^ and inflammation.^8^

This protein is widely distributed in most peripheral organs, including the heart, kidney, lungs, nasal epithelium, and adrenal glands with the highest expression in steroid producing tissues. In the central nervous system, TSPO is not homogeneously expressed: some cerebral regions, such as cerebellum and hippocampus, show higher levels of TSPO and, generally, its expression in the white matter is higher than in the gray one;^2^ the cellular origin of TSPO has been identified in astrocytes in the brain. TSPO is also found in retina,^9^ where it is highly expressed in Muller cells and retinal pigment epithelium (RPE); it is also expressed in cerebral as well as retinal microglia and in dopaminergic neurons.^10^

TSPO expression has been found to be altered in a variety of human diseases, mainly in neurodegenerative and neuroinflammatory diseases,^11^ including Parkinson’s disease, Huntington’s disease, dementia, amyotrophic lateral sclerosis, Alzheimer’s disease, multiple sclerosis,^12^ and in some neuropsychiatric diseases.^13^

TSPO has been now widely recognized as an attractive druggable target, as TSPO ligands showed protective effects in different in vitro and in vivo models of neuropathologies with inflammation-related features.^14^ The beneficial effects exerted by TSPO ligands against neuroinflammation are mediated by the modulation of several biological processes, such as the regulation of neurosteroids’ production, cytokine release, and radical oxidative species metabolism.^15^

In addition, the evidence of TSPO level upregulation in microglia and astrocytes during an injury in the CNS leads to consider TSPO as a suitable diagnostic biomarker for positron emission tomography (PET) imaging studies in several CNS pathologies.^16^ Numerous PET radioligands that target TSPO have been developed for imaging inflammatory progression in the brain.^17^

Classical synthetic TSPO ligands are the 4′-chloro-derivative of diazepam (Ro5–4864) and the isoquinoline carboxamide PK11195,^18,19^ which rendered them capable of inhibiting the secretion of pro-inflammatory cytokines, the proliferation of monocytes and to reduce the lipopolysaccharide (LPS)-induced upregulation of inflammatory factors, such as cyclooxygenase (COX)-2, and the tumor necrosis factor (TNF)-α in cultured rodent and human microglia.^14^

In the last decades, several classes of TSPO ligands endowed with anti-inflammatory and neuroprotective effects in both in vitro and in vivo models have been identified.^14,20−23^ In particular, TSPO ligands are proposed as therapeutic tools for Alzheimer’s disease,^21^ Parkinson disease,^10^ multiple sclerosis,^24^ neuropathic pain,^25^ and anxiety disorders.^26^

From a mechanistic point of view, it has been suggested that TSPO ligands could be effective in neuroprotection by boosting mitochondrial function and by modulating endogenous production of neurosteroids.^27^ Specifically, TSPO binds cholesterol and, together with steroidogenic acute regulatory protein (StAR), promotes its translocation from the outer to the inner mitochondrial membrane. Once in the mitochondria, cholesterol enters the first step of steroid synthesis, where it is converted by the cytochrome P450 side chain cleavage enzyme (P450ssc) into pregnenolone, which is the precursor of all neurosteroids,^28^ many of which are known to exert neuroprotective effects.

Starting from 2004,^29^ some of us developed a class of potent and selective TSPO ligands, *N*,*N*-dialkyl-2-arylindol-3-ylglyoxylamides (PIGAs),^30,31^ endowed with nanomolar/subnanomolar TSPO affinity. A number of PIGAs showed the ability to stimulate steroid production in rat C6 glioma cells through their binding to TSPO, and some of them exerted in vivo anxiolytic/nonsedative effects (elevated plus-maze (EPM) test in rats)^30,32,33^ and were able to control inflammatory mechanisms in a mice model of human primary progressive multiple sclerosis.^34^

In addition, a number of PIGAs promoted in vitro the protection of human astrocytes from oxidative stress and inflammation, by decreasing the pro-inflammatory enzymes’ expression (inducible nitric oxide synthase, iNOS and COX-2) and by increasing the release of brain-derived neurotrophic factor (BDNF) in human microglial cells (C20 and HMC3).^28^ All these effects were shown to be mediated by the production of neurosteroids.^28,32,35,36^

Based on these previously described pro-survival activities of PIGAs, the aim of the present study was to assess TSPO expression in 661 W photoreceptor cell line and to evaluate the potential of targeting TSPO for the therapeutic treatment of retinal neurodegeneration. For this purpose, the above-mentioned cell line was exposed to a toxic inflammatory insult (i.e., LPS 10 μg/mL), as an in vitro model of retinal neurodegeneration, and the protective effect of a number of selected PIGAs was evaluated. Results from our studies demonstrated that (i) TSPO was expressed in 661 W photoreceptor cell line, in both physiological and pathological conditions, supporting TSPO expression in retinal photoreceptors; (ii) the inflammatory process triggered by LPS caused a failure of TSPO insertion in mitochondrial membranes, which was restored by treatment with the tested ligands, while the total TSPO protein levels were unaltered; (iii) the selected TSPO ligands exerted a neuroprotective action by modulating the inflammatory and apoptotic responses after LPS insult.

Overall, the data obtained here open a new therapeutic perspective for slowing down the progression of retinal neurodegenerative diseases, such as retinitis pigmentosa (RP), by counteracting the inflammatory processes.

## Results and Discussion

It is reported in the literature that XBD173, a TSPO ligand, is able to reduce microglial activation by reducing the levels of pro-inflammatory species such as interleukin-6 (IL6) and the chemokine (C–C motif) ligand 2 (CCL2), thus decreasing the inflammatory processes, including those related to retinal degeneration.^37,38^ The present study aims to evaluate the potential of targeting TSPO expressed into retinal neurons to produce an anti-inflammatory effect; for this purpose, the anti-inflammatory activity of PIGA ligands was evaluated in an in vitro photoreceptor-like model, the 661 W cell line, after LPS-induced damage.

**Figure 1:**
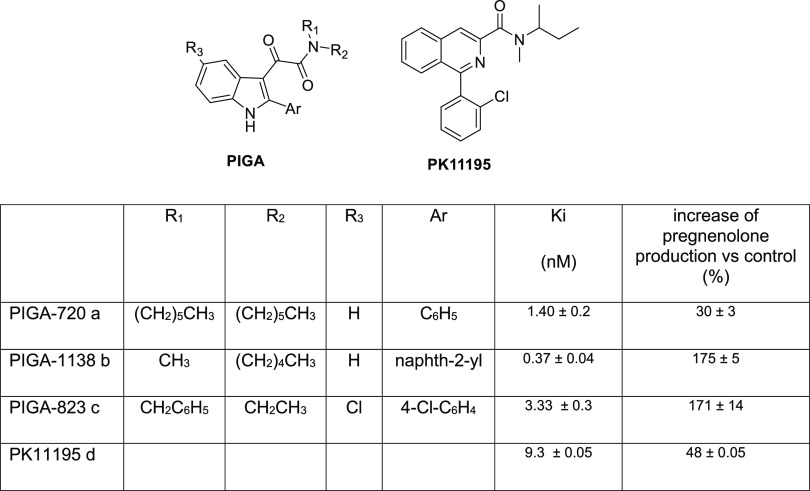
General structure of *N*,*N*-dialkyl-2-phenylindol-3-ylglyoxylamides PIGAs and PK11195. ^a^From ref (29). ^b^ From refs (31, 50). ^c^ From ref (30). ^d^ From ref (30).

A number of TSPO selective ligands were selected from our in-house collection of PIGAs, a wide library developed by some of us since 2004.^29−31^ Specifically, three PIGAs endowed with low nanomolar/subnanomolar TSPO affinity and moderate to high pro-steroidogenic activity were selected, as reported in Figure 1. In detail, PIGA-823 and PIGA-1138 represent prototypes of highly steroidogenic compounds, showing a 171 and 175% increase of pregnenolone production versus control, respectively, while PIGA-720 displays moderate pro-steroidogenic activity (30% vs control) (Figure 1). The classic TSPO ligand PK11195 (Figure 1) was used as the reference standard.

**Figure 2:**
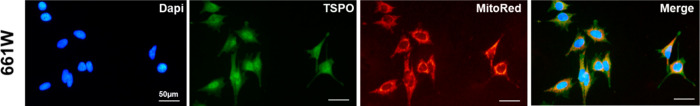
TSPO detection and localization in 661 W nondamaged and untreated cells. The images show the staining for TSPO protein (anti-TSPO antibody, in green) and for a mitochondria-specific marker (MitoRed, in red). Cell nuclei (DAPI) are stained in blue. The last image shows the colocalization (yellow) between TSPO protein and mitochondria. Scale bars: 50 μm.

As a first assessment, the expression of TSPO in the cell model used was evaluated as, to the best of our knowledge, it has been never reported in the literature. Figure 2 shows the presence of TSPO (green staining) in photoreceptor-like 661 W cells, and the merge obtained with a mitochondria-specific marker (red staining) shows that the protein is localized at the level of the mitochondria itself (yellow marking).

**Figure 3:**
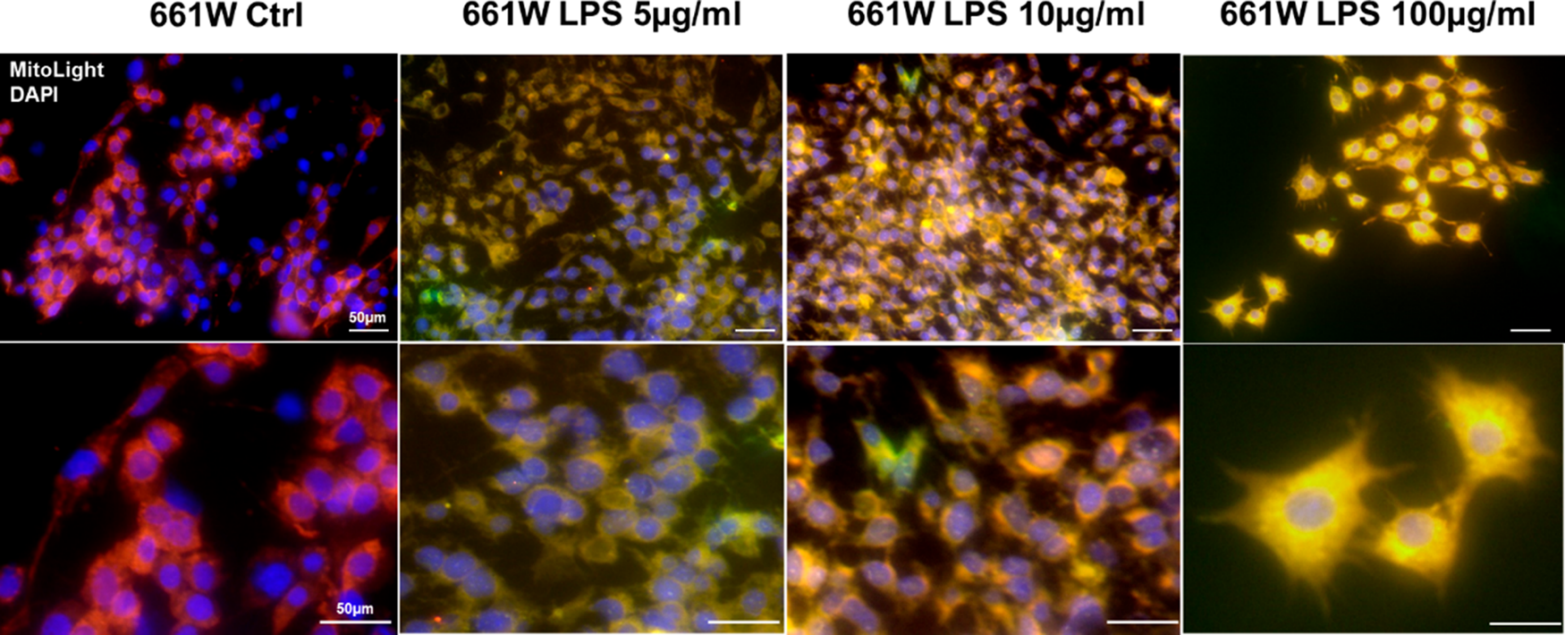
LPS-induced inflammatory damage in 661 W cell line. The images show the cells treated with increasing concentration of LPS and stained with a mitochondrial marker (MitoLight) that is able to change color from red to green in a membrane potential-dependent manner. The red signal indicates that the mitochondrial membrane is intact (the dye is localized in the mitochondria, living cells), while the presence of a green/yellow signal indicates a change in membrane potential, and the dye remains in the cytosol where it aggregates (apoptotic cell). In blue (DAPI) are labeled the cell nuclei. Scale bars 50 μm.

Then, the LPS damage in the 661 W cell line was validated. Figure 3 shows that increasing the concentration of LPS from 5 to 100 μg/mL resulted in a change from red (living cells) to green (apoptotic cells) in the staining obtained with the mitochondrial marker MitoLight; this dye can change color according to its state of aggregation and in a membrane potential-dependent manner. Based on the results obtained and shown in Figure 3, we identified the ideal concentration to obtain an adequate stress for the 661 W cells, which was 10 μg/mL LPS.

**Figure 4:**
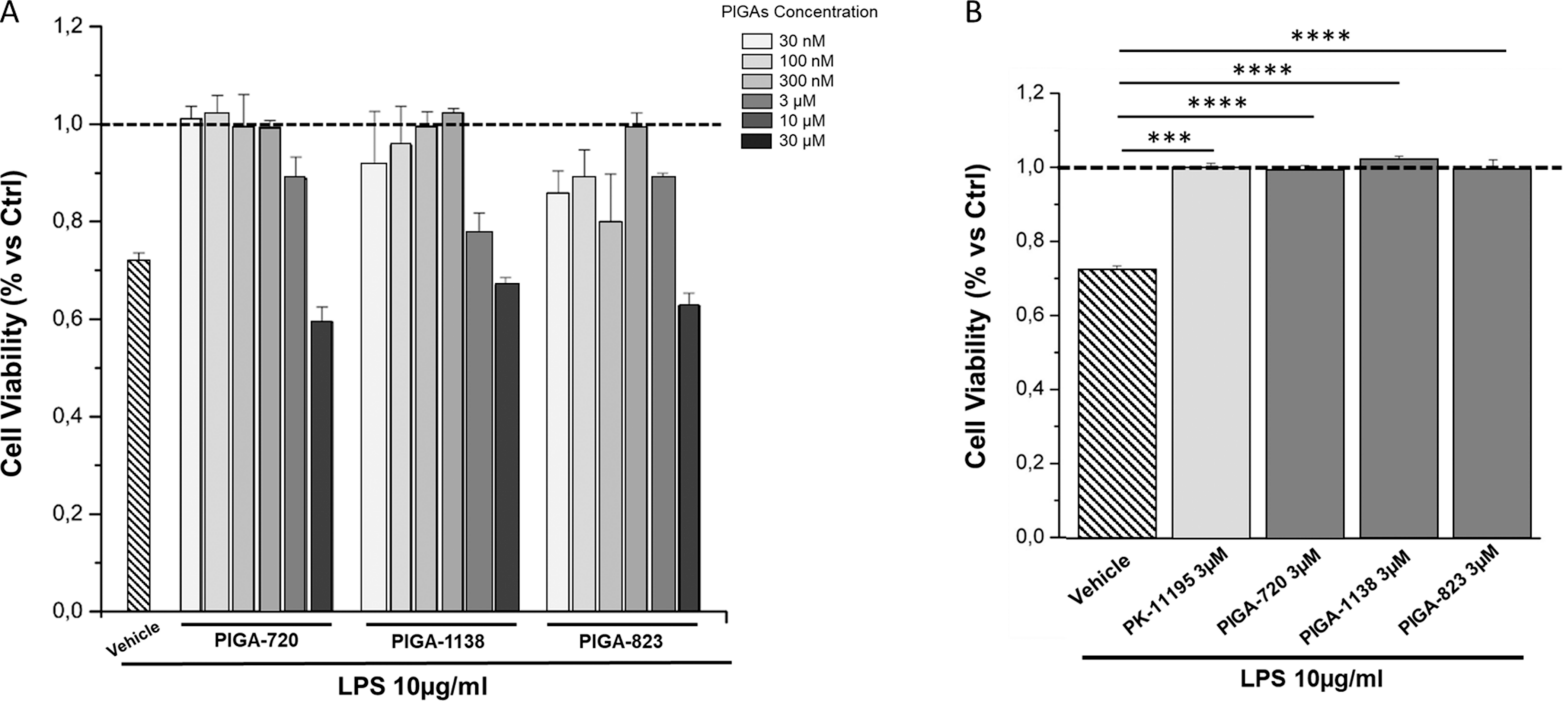
Treatment with PIGA ligands protects 661 W photoreceptor-like cells from LPS-induced damage. (A) Bar graph shows different treatment concentrations (30 nM–30 μM) for each compound tested; the slanted line bar indicates LPS-damaged cells in the absence of PIGAs (vehicle), while the dashed line indicates the reference value of nondamaged and untreated cells (control—ctrl). (B) Bar graph shows the efficacy of the tested ligands in protecting 661 W cells, from LPS-induced damage, at the dose chosen for all experiments (3 μM) in comparison with known TSPO ligand, PK-11195 (3 μM, light gray bar) and with vehicle (slanted line bar). The dashed line indicates the reference value of nondamaged and untreated cells. Values in the graph indicate % viability as the mean ± SE obtained from a *n* = 5 of independent experiments; statistics: Student’s *t*-test followed by Bonferroni’s post-test *p ≤ 0.05, ***p ≤ 0.001, ****p ≤ 0.0001.

Actually, the damage produced by 10 μg/mL of LPS allowed us to obtain a cell mortality of about 25% (Figure 4A, bar relative to stressed and untreated cells, DMSO), that was adequate to evaluate the potential protective effect of PIGAs (Figure 4). Figure 4 shows how treatment of 661 W cells with PIGAs preserved the cells from death processes by restoring viability values to those obtained for unstressed and untreated cells, which was set to 1.0. The protective effect reached its maximum at very low concentration (30 nM) for PIGA720. Conversely, for PIGA1138 and PIGA823, the protective activity improved with concentration up to 3 μM. Actually, at the concentration of 3 μM, all tested PIGAs were able to restore cell viability as in the unstressed and untreated control and analogous to that obtained with the reference PK11195, tested at the same concentration (3 μM). Only for PIGA1138, a significant higher percentage of viability was obtained compared to PK11195, suggesting a possible pro-proliferative activity of this molecule (Figure 4B).

It should be outlined that the cytoprotective effect exerted by PIGAs does not directly correlate with their pro-steroidogenic properties (see Figure 1). Although these two parameters were evaluated in different cell lines (661 W cells for cell viability and C6 glioma cells for pregnenolone production), it is reasonable to assume that other biological pathways/mechanisms, not related to steroids’ synthesis, might concur to the effect observed in photoreceptor-like cell lines.

For all PIGAs tested, increasing the concentrations over 3 μM, and up to 30 μM, produced a reduction of viability to values even lower than those obtained for unstressed and untreated cells (Figure 4A). This trend could be ascribed to a nonspecific cytotoxic effect that arises as a consequence of ligands’ high concentration.

Based on the results obtained, 3 μM was chosen as the best concentration of PIGAs to be used for further experiments.

**Figure 5:**
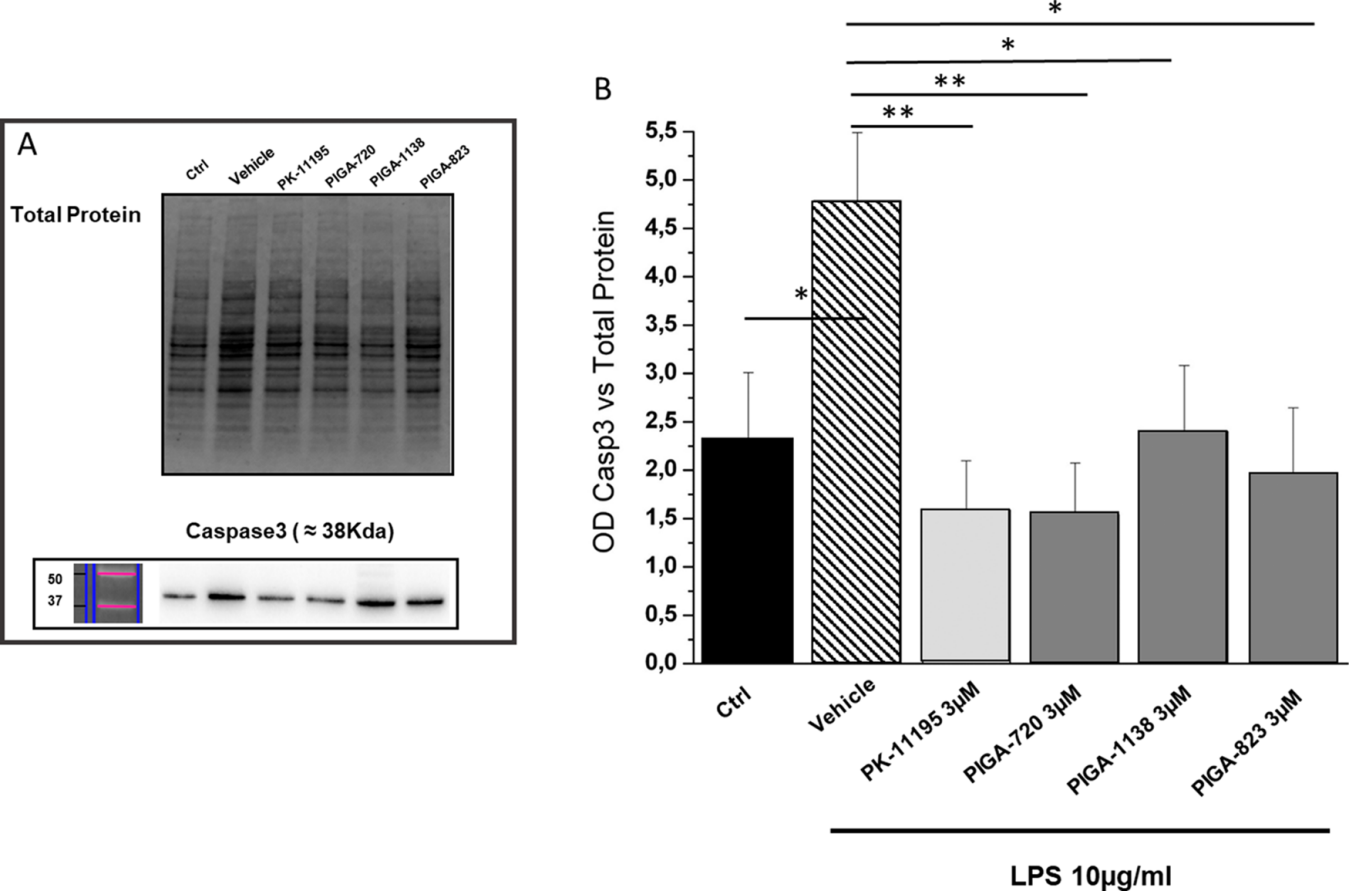
PIGAs treatment reduces caspase3 levels in LPS-stressed cells. (A) Representative example of western blot obtained by loading all samples under analysis; (B) bar graph showing caspase-3 protein levels (measured by optical densitometry, OD) versus total protein content (total protein): black bar (ctrl) indicates nondamaged and untreated cells; slanted line bar (vehicle) indicates damaged and untreated cells; light gray bar indicates the treatment with known TSPO ligand, PK-11195 (3 μM); gray bar indicates the treatment with the different PIGA compound (3 μM). Values in the graph indicate the mean ± SE obtained from *n* = 5 of independent experiments; statistics: Student’s t-test followed by Bonferroni’s post-test *p ≤ 0.05, **p ≤ 0.01.

To investigate the mechanisms underlying the protective effect exerted by these compounds, we assessed the protein levels of the apoptotic marker caspase-3 by western blot. We found that all PIGAs completely blocked the LPS-induced enhancement of caspase-3 expression (Figure 5).

**Figure 6:**
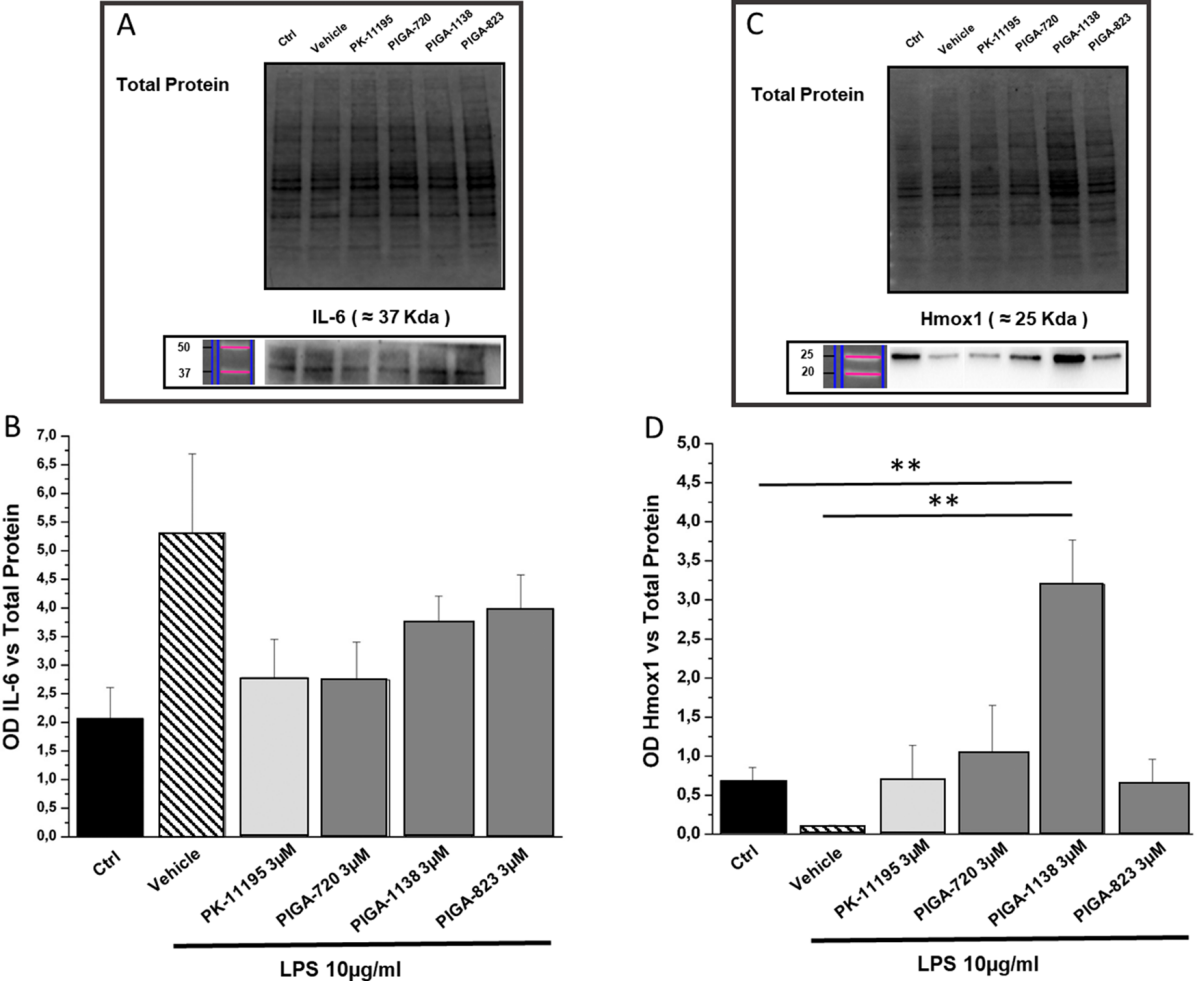
PIGAs treatment modulates the levels of inflammatory markers in LPS-stressed cells. (A, C) Representative example of western blot obtained by loading all samples under analysis respectively for IL-6 and Hmox1 analysis; (B,D) bar graphs showing respectively IL-6 and Hmox1 protein levels (measured by optical densitometry, OD) versus total protein content (total protein): black bar (ctrl) indicates nondamaged and untreated cells; slanted line bar (vehicle) indicates damaged and untreated cells; light gray bar indicates the treatment with known TSPO ligand, PK-11195 (3 μM); gray bar indicates the treatment with the different PIGA compound (3 μM). Values in the graph indicate the mean ± SE obtained from a *n* = 5 of independent experiments. Statistics: Student’s t-test followed by Bonferroni’s post-test **p ≤ 0.01.

We also examined the protein levels of two of the best-known markers of inflammation, IL-6 and heme oxygenase 1 (Hmox-1). The immunoblot in Figure 6 shows that TSPO ligands counteracted the LPS-driven overexpression of IL-6, restoring its levels to control values. Moreover, PIGAs were able to prevent the LPS-induced decrement of Hmox-1 expression, an enzyme with anti-inflammatory properties. Of note, PIGA1138 showed a particular efficacy in inducing an increase of Hmox-1 that could be somehow correlated with a modulation of gene expression. These results suggest that TSPO ligands are able to break the inflammatory processes, while promoting the anti-inflammatory pathways. Our results are in line with the previously demonstrated anti-inflammatory activity of TSPO ligands, likely due, at least partially, to their role in increasing the de novo synthesis of steroidogenic molecules derived from cholesterol (e.g., pregnenolone).^28^

**Figure 7:**
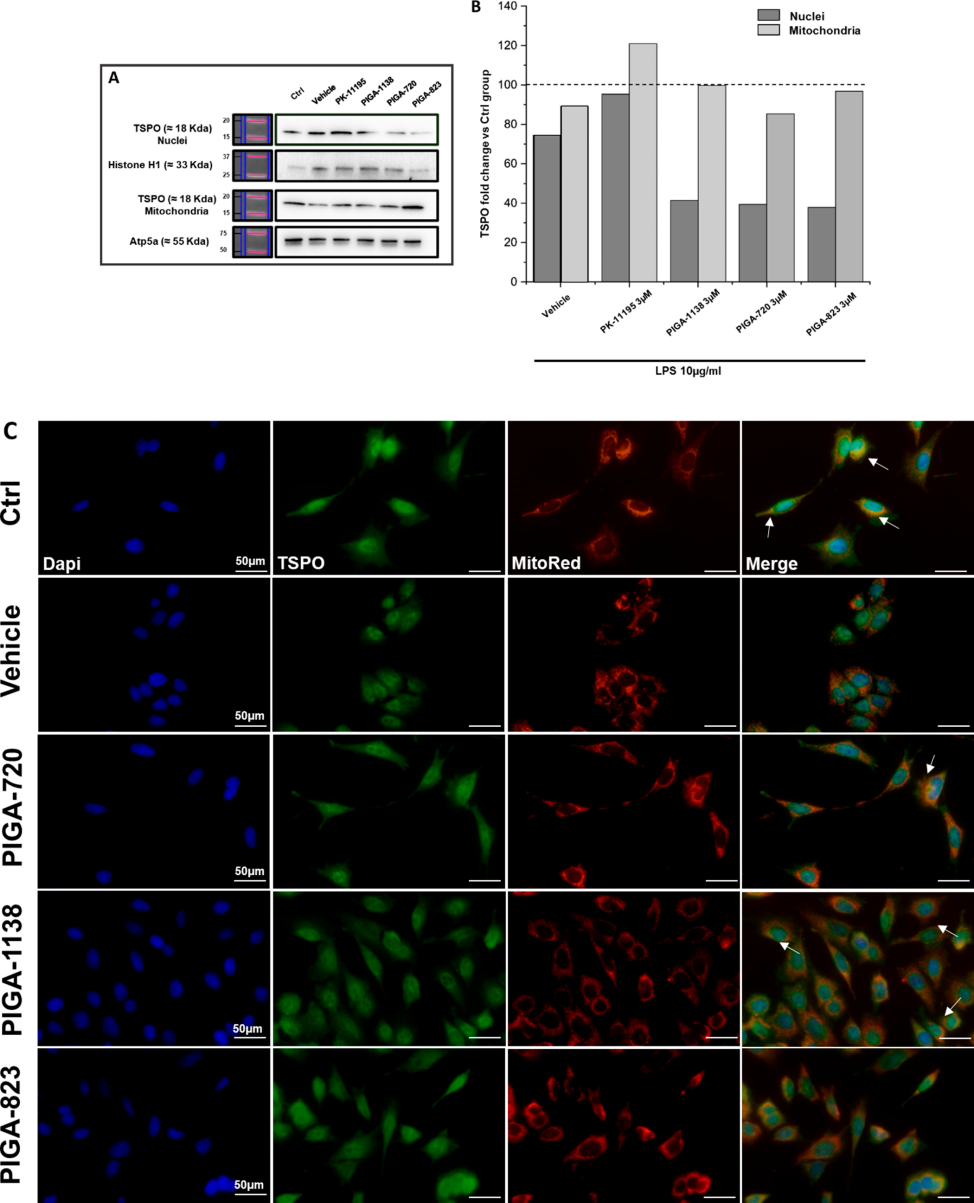
Treatment with PIGAs restores the correct localization of TSPO at the level of the mitochondrial membrane. (A,B) Representative immunoblots of TSPO. Bar graphs showing TSPO protein fold change levels versus the control group: dashed line bar indicates nondamaged and untreated cells; gray bar and light gray bar indicate, respectively, the nuclei and the mitochondria levels of TSPO of the treatment groups. Values in the graph indicate the percentage in terms of fold increase obtained from a *n* = 3 of independent experiments, respectively, for nuclei and mitochondria fractions compared to the control group. (C) Images show the staining for TSPO protein (anti-TSPO antibody, in green) and for a mitochondria-specific marker (MitoRed, in red). Cell nuclei (DAPI) are stained in blue; the arrows in the marge panels indicate the colocalization of TSPO and mitochondria. Scale bars 50 μm.

The literature on TSPO reports that this transporter is usually overexpressed in neuroinflammation;^20^ however, recent studies demonstrated a cell-specific increase in TSPO, and that this increase at the cell surface did not correlate with an increase of the intracellular concentration of the protein or of its mRNA levels.^39^ The data reported here indicate that in our 661 W cell model of LPS-induced neuroinflammation, there was no increase in total TSPO protein levels (Figure S1), but the transporter changed its localization in the cell by altering the ratio of the percentage of its expression at the nuclear and mitochondrial levels (Figure 7A,B). In Figure 7C, it can be observed that cells treated with PIGAs show a staining pattern more similar to the unstressed/untreated control cells (CTRL), where TSPO is localized at the level of the mitochondrial membrane, whereas in the cells stressed and treated with vehicle alone (DMSO), the colocalization was completely lost, and TSPO localized mainly at the peri-nuclear level (vehicle alone treated cell, DMSO, green staining). This shift of localization could lead to a loss of the protein’s ability to bind to their ligands, as demonstrated by Fan and collaborators in a previous study.^40^ Treatment with PIGAs completely restored the physiological localization of TSPO at the level of mitochondria (Figure 7C, arrows), likely preserving mitochondrial bioenergetics and avoiding the triggering of cell programmed death processes.^41^ Our data demonstrate, for the first time, the TSPO expression in photoreceptor-like cells and lead us to hypothesize a possible direct action of TSPO in the control not only of inflammation but also of apoptosis and proliferation of this cell line.^39^

**Figure 8:**
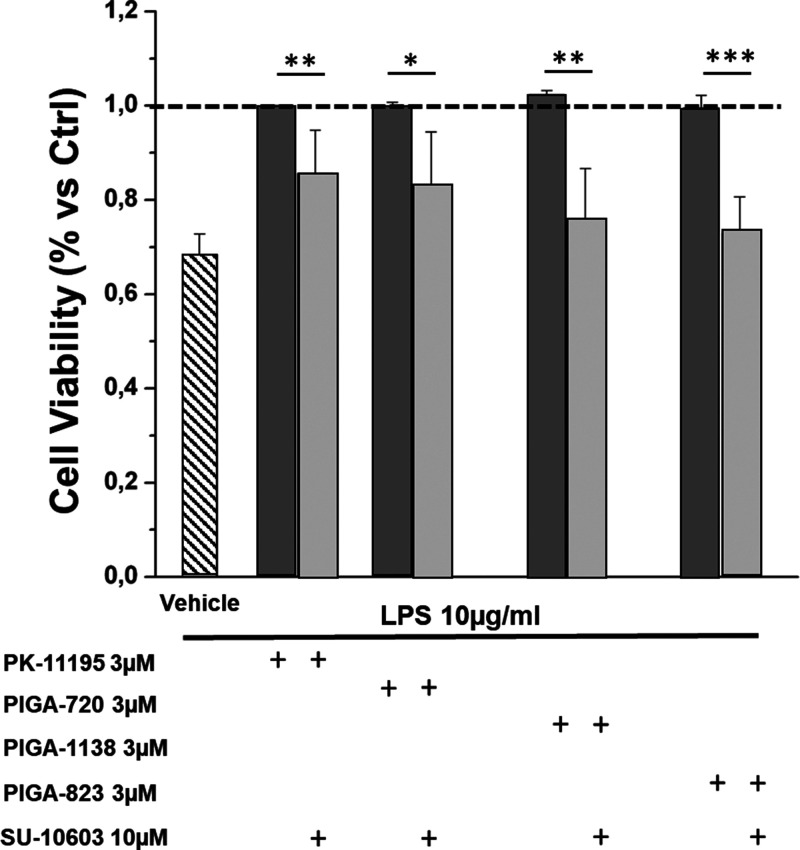
The protective effect of TSPO ligands, PIGAs, is mediated by an increase in the production of anti-inflammatory steroids. Bar graph shows cell viability, expressed as percentage compared with nondamaged and untreated cells (dashed line bar), following combined treatment with PIGAs (3 μM) and pregnenolone synthesis inhibitor SU10603 (10 μM) (light gray bars) compared to PIGAs (3 μM) alone treated cells (dark gray bars). Slanted line bar represents damaged and untreated cells (vehicle). Values in the graph indicate % viability as the mean ± SE obtained from a *n* = 5 of independent experiments; statistics: Student’s t-test followed by Bonferroni’s post-test *p ≤ 0.05, **p ≤ 0.01, ***p ≤ 0.001.

Finally, to assess whether the protective activity of TSPO ligands was, at least in part, mediated by the production of anti-inflammatory steroids, 661 W cells were cotreated with the ligands (3 μM) and the inhibitor of pregnenolone synthesis (10 μM), SU10603 (inhibitor of 17α-hydroxylase/C17–20 lyase (P450c17 or CYP17A1)), which prevents the conversion of pregnenolone into deidroepiandrosterone (DHEA).^36,42^ From the bar graph shown in Figure 8, it is possible to observe a significant reduction in the protective activity of all TSPO ligands, evidencing, in particular for PIGA823 and PIGA1138 (highly steroidogenic), cell viability values comparable to those of the stressed/untreated group (DMSO), indicating an almost total suppression of their efficacy. The residual activity maintained by PIGA720 could be ascribed to the activation of other pathways, such as an increase of cell proliferation.

## Conclusions

The 18 kDa TSPO is overexpressed at the level of active microglia and in astrocytes at the site of neuronal damage and different TSPO ligands display significant neuroprotective activities in animal models of neurodegeneration.^10,43,44^ Here, we demonstrated, for the first time, the presence of TSPO in 611 W cells, an in vitro photoreceptor-like model; furthermore, the data collected in our study showed that a LPS-mediated inflammatory insult did not cause an alteration in the intracellular protein levels of TSPO, but changed its localization from mitochondrial to peri-nuclear area. The TSPO ligands tested in this study significantly protected 661 W cells from LPS-induced damage by mediating both anti-apoptotic and anti-inflammatory actions and by preserving the correct localization of TSPO at the mitochondrial membrane. The ability of PIGAs to maintain mitochondrial localization of TSPO could explain the neuroprotection in terms of increased cellular viability. In fact, it is reported in the literature that the transfection of TSPO is able to preserve cells from UV-induced apoptosis by reducing the activity of caspase-3 and the production of ROS;^45^ furthermore, PIGAs, as other TSPO ligands, in addition to the steroidogenic activity, could also increase ATP levels and stabilize the mitochondrial membrane potential preventing the onset of apoptotic processes.^46^

In a recent study,^47^ the authors reported the synthetic progestin “Norgestrel” to exert neuroprotective and anti-inflammatory action in the rd10 animal model, preserving retinal morphology and functions; this evidence strongly supports the role of pregnenolone in reducing neurodegeneration in this animal model. In addition, in another recent study,^48^ it was shown that TSPO is also expressed at the level of the retina and RPE and that in rd10, TSPO is overregulated up to one year of age. These findings, together with our results, lead us to hypothesize that TSPO ligands might represent useful tools to reduce the inflammation occurring in neurodegenerative retinal diseases, such as RP, by preserving photoreceptors from death through hormone modulation via pregnenolone.

In most RP patients, the primary mutation, which causes degeneration, is localized at the level of the rods and the secondary one is the death of the cones that occurs due to multiple effects that add up to each other, including inflammation. This gives rise to the need of identify possible pathways involved in the inflammatory process that can be used in all individuals with RP to slow the secondary degeneration of cones. Although further studies are needed also in in vivo models of RP, it is possible to speculate that the anti-inflammatory effects obtained in our cell model may translate, in vivo, into prolonged visual function due to a slowing of cone degeneration and death. Accordingly, targeting TSPO might represent a successful strategy to put in place a novel and viable therapeutic strategy to slow down the degenerative process that leads to the inexorable loss of vision.

## Materials and Methods

### General Chemistry Direction

PIGA720,^29^ PIGA823,^30^ and PIGA1138^31^ were prepared as previously described.

### Cell Culture

The 661 W photoreceptor cells were supplied by Dr. Muayyad Al-Ubaidi (University of Oklahoma Health Sciences Center). Cells were grown in Dulbecco’s modified Eagle’s medium—high glucose (DMEM) with 10% fetal bovine serum (FBS), 1% penicillin/streptomycin, and 1% l-glutamine at 37 °C in a 95% O_2_ and 5% CO_2_ humidified atmosphere. The material used for cell cultures was purchased from Sigma-Aldrich (Merck, Darmstadt, Germany).

### Drug Stock Preparation

Lipopolysaccharides (LPSs) (from *Escherichia coli*) PK-11195 and SU-10603 were purchased from the Sigma-Aldrich, while the compounds PIGA-720, PIGA-1138, and PIGA-823 were synthesized as described above. LPS stock solution (1 mg/mL) was prepared in physiological saline and then diluted to working concentrations in DMEM. Stock solutions of PIGA-720, PIGA-1138, PIGA-823, PK-11195, and SU-10603 (10 mM) were prepared in DMSO and then diluted to working concentrations in DMEM.

### Cell Viability

Cell vitality was tested by using CellTiter 96 Aqueous—One Solution Reagent (Promega, WI, USA). Cells were seeded in a 96-well plate with a density of 10^4^ cells/well. The next day, they were starved (DMEM without l-glutamine and FBS) for 4 h and then treated with LPS (10 μg/mL) together with vehicle (DMSO) or with the compounds at different concentrations for 24 h. At the end of the treatment, cells were treated with the one solution reagent and after 2 h, the viability was measured by the Ensight instrument.

### Immunostaining

Cells were seeded into an 8-well chamber slide at a density of 10^4^ cells/well. The next day they were starved for 4 h. To assess the damage caused by different concentrations of LPS (5, 10, and 100 μg/mL) for 24 h, cells were stained with MitoLight Mitochondrial Apoptosis Detection Kit (Millipore, USA) for 15 min at 37°, before fixation with 2% PFA for 15 min followed by three washes with PBS. After washes, cell nuclei were stained with DAPI (Sigma-Aldrich) 1:5000 diluted in PBS. MitoLight partitions differently in healthy cells than in apoptotic cells. In healthy cells, the dye accumulates and aggregates in the mitochondria, emitting a bright red fluorescence. In apoptotic cells, with altered mitochondrial membrane potential, the dye in its monomeric form remains in the cytoplasm, with a green fluorescence, providing ready discrimination between apoptotic and nonapoptotic cells.

To evaluate the efficacy of the compounds at the chosen concentration (3 μM) with LPS damage (10 μg/mL) for 24 h, cells were stained by adding 50 nM MitoRed kit (Sigma-Aldrich) diluted in DMEM for 1 h at 37°, before fixation with 2% PFA for 15 min followed by three washes with PBS. Then the cells were permeabilized and blocked with a PBS solution containing 2% BSA and 0.3% Triton X100 for 45 min at room temperature and then incubated overnight at 4 °C with primary antibody diluted in a PBS solution containing 2% BSA and 0.03% Triton X100 (anti-TSPO 1:200—Invitrogen, USA). The day after, cells were washed with PBS and incubated for 2 h with an anti-rabbit goat IgG conjugated with an Alexa Fluor 488 dye (Thermo Fisher Scientific, 1:500), in a PBS solution containing 2% BSA. After washes, cell nuclei were stained with DAPI (Sigma-Aldrich) 1:5000 diluted in PBS. Coverslips were mounted with Vectashield on a microscope support and imaged with a Nikon Ni-E fluorescence microscope equipped with a DS-Ri2 camera using a 60× oil objective.

### Western Blot

Cells were seeded in 6 cm Petri dishes at a density of 3 × 10^5^ cells. The next day, they were starved for 4 h and later treated with the compounds, or vehicle alone, and LPS. After 24 h, cells were lysed by adding 200 μL of RIPA buffer (150 mM NaCl, 50 mM Tris–HCl pH 8, 1% Igepal, 0.5% Na-deoxycholate, 0.1% SDS; and protease inhibitors—1 μM Orthovanadate and 0.1 mg/mL PMSF). The cellular suspension was sonicated and kept in ice for 30 min. Then they were centrifuged at 12,000 rpm for 30 min at 4 °C. Protein quantification of the cell lysates was performed by using Bradford assay. 30 μg of total protein from each condition were mixed with 2× Laemmli solution and loaded into precast 4–20% polyacrylamide gels (mini-PROTEAN TGC gel, Bio-Rad). Then, the gel was activated by using ChemiDoc XRS+ (Bio-Rad, California) instrument, and the separated proteins were transferred to PVDF membranes (Trans-Blot Turbo PVDF Transfer packs, Bio-Rad).

The membrane was incubated with EveryBlot Blocking Buffer solution for 5 min and then for 1 h at room temperature with a primary antibody in EveryBlot. Next, the membrane was washed 5 × 5 min with t-TBS (Tris-buffered saline solution added with 0.05% Tween20). The membrane was incubated again for 1 h at room temperature with the secondary antibody in EveryBlot, washed 5 × 5 min with t-TBS, and then the immunoblot signal was detected with the enhanced chemiluminescence substrate detection system (LuminataTM Forte Western HRP Substrate, Millipore). The chemiluminescent images were acquired by ChemiDoc XRS· + (Bio-Rad, California) instrument. Proteins were acquired to normalize intensity of the investigated band with total protein.^49^ Densitometry was undertaken using Bio-Rad ImageLab software.

For mitochondrial fractionation, cells were seeded in a 15 cm Petri dishes at a density of 5 × 10^6^ cells. The next day, they were starved for 4 h and later treated with the compounds, or vehicle alone, and LPS. After 24 h, cells were lysed by adding 3 mL of mitochondrial isolation buffer (sucrose 0.32 M, EDTA 1 mM, Tris–HCl 10 mM pH 7.4). The cellular suspension was sonicated and then centrifuged at 600*g* for 10 min at 4 °C. The supernatant was collected and centrifuged again at 10,000*g* for 25 min at 4 °C, while the pellet was saved because that is where the nuclei are. At the end of the second centrifugation, the pellet obtained is the mitochondrial pellet. 50 μL of RIPA buffer was added to both pellets, and then the same protocol as described above was followed.

Antibodies and dilutions used are listed below: mitochondria fraction western blot cocktail mouse (Abcam, 1:250); Anti-TSPO Rabbit (Invitrogen, 1:500); Anti-Caspase3 Rabbit mAb (Millipore, 1:1000); anti-HMOX1 Mouse mAb (Invitrogen, 1:500); anti-IL6 Mouse mAb (Invitrogen, 1:1000); Anti-Rabbit IgG HRP (Sigma-Aldrich, 1:5000); and Anti-Mouse IgG HRP (Sigma-Aldrich, 1:5000).

### Statistical Analysis

The Origin Lab 8.0 program (MicroCal, Northampton, MA, USA) was used for data analysis and graphic presentation. All data are presented as the means ± SEMs. Statistical analyses were performed by using Student’s t-test followed by Bonferroni post-test as indicated in each graphic. The *p*-value ≤ 0.05 was considered to be statistically significant.

## Supporting Information Available

Western blot of whole lysate for TSPO protein (PDF)
