# Supplementary material for: Targeting TSPO Reduces Inflammation and Apoptosis in an In Vitro Photoreceptor-Like Model of Retinal Degeneration
Source: ACS Chem Neurosci. 2022 Oct 27;13(22):3188–97. doi: 10.1021/acschemneuro.2c00582 (PMC9673150; doi:10.1021/acschemneuro.2c00582)
Supplement: Supplementary file 1 — cn2c00582_si_001.pdf [file cn2c00582_si_001.pdf]

Supporting Information

Targeting TSPO reduces inflammation and apoptosis in an *in-vitro* photoreceptor-like model of retinal degeneration

Francesca Corsi[1], Emma Baglini[1], Elisabetta Barresi[1£], Silvia Salerno[1], Chiara Cerri[1], Claudia Martini[1], Federico Da Settimo Passetti[1], Sabrina Taliani[1], Claudia Gargini[1] and Ilaria Piano[1£]

[1]Department of Pharmacy, University of Pisa, 56126 – Italy

[£]Corresponding Authors: elisabetta.barresi@unipi.it; ilaria.piano@unipi.it

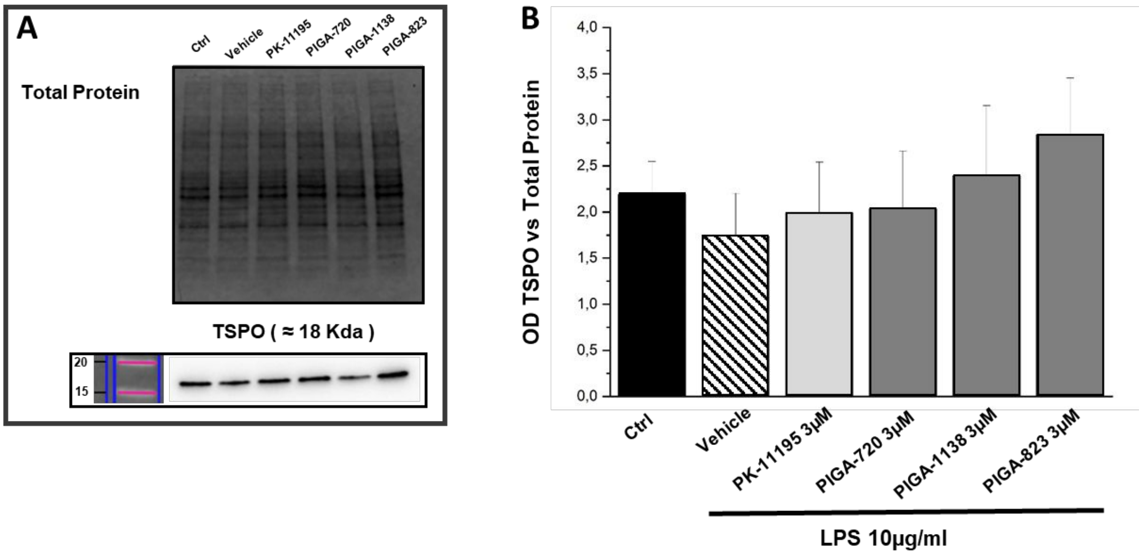

Figure S1. (A) Representative example of western blot obtained by loading all samples for TSPO analysis. **(B)** Bar graphs showing TSPO protein levels (measured by optical densitometry, OD) versus total protein content (Total Protein): black bar (Ctrl) indicates non-damaged and untreated cells; slanted line bar (vehicle) indicates damaged and untreated cells; light gray bar indicates the treatment with known TSPO-ligand, PK-11195 (3 µM); gray bar indicates the treatment with the different PIGA compound (3 µM) Values in the graph indicate the mean ± SE obtained from a n=5 of independent experiments. No significant results were obtained.
